# Frontiers in the COVID-19 vaccines development

**DOI:** 10.1186/s40164-020-00180-4

**Published:** 2020-09-03

**Authors:** Ehtisham Ul Haq, Jifeng Yu, Jiancheng Guo

**Affiliations:** 1 School of Basic Health Sciences, Zhengzhou University, No. 100, Science Avenue, Zhengzhou, 450001 China; 2 The First Affiliated Hospital of Zhengzhou University, #1 East Jianshe Road, Zhengzhou, 450052 China; 3 Academy of Medical and Pharmaceutical Sciences of Zhengzhou University, #40 Daxue Road, Zhengzhou, 450052 China; 4 Precision Medicine Center of Zhengzhou University, #40 Daxue Road, Zhengzhou, 450052 China; 5 Department of Molecular Pathology, The Second Affiliated Hospital, Zhengzhou University, #2 Jingba Road, Zhengzhou, 450052 China

**Keywords:** COVID-19, Vaccine development, RNA and DNA vaccine

## Abstract

Novel corona virus caused pneumonia first reported in December, 2019 in Wuhan, China was later named COVID-19. Due to its special pathogenicity, COVID-19 transmitted with high speed beyond borders and has significantly affected normal life. Currently, no specific drugs, treatment or vaccines are available. Vaccine development for COVID-19 is a highly complex process involving viral genomic studies, identification of target for vaccine, vaccine design, manufacturing, storage and distribution, preclinical and clinical safety and efficacy studies. The high levels of efforts and global collaboration at this scale is unprecedented. The World Health Organization (WHO) has documented 160 different COVID-19 vaccine candidates as of July 13, 2020 with 26 currently on clinical evaluation while 137 vaccines on preclinical evaluation. COVID-19 vaccine efforts mark the first use of mRNA-type vaccines ever evaluated. Numerous research organizations have successfully initiated clinical evaluation of COVID-19 vaccines. This review aims to summarize the advances and challenges for COVID-19 vaccines development.

To the editor,

On December 31, 2019, novel corona virus caused pneumonia was first reported in Wuhan, China. The pathogen was soon identified as a novel corona virus from unknown origin and then was named as “corona virus of 2019” or “COVID-19”. With a rapid spread of the virus, WHO declared a global pandemic on March 11, 2020. According to WHO, as of July 14, 2020, almost all countries in the world have been affected with 12,768,307 confirmed cases and 566,654 confirmed deaths due to COVID-19 (https://covid19.who.int/). Its highly infectious and asymptomatic transmission characteristics have made it to a pandemic in a short time [1]. Vaccines are an essential countermeasure urgently needed to control the pandemic.

2-dimension and 3-dimension studies demonstrated COVID-19 virus as RNA stranded virus, surrounded by membrane (M) protein, envelope (E) protein, and the spike (S) structural protein. Genome of virus is highly packed inside nucleocapsid (N) protein which is enveloped by M, E and S protein [2]. Five nonstructural proteins including ORF1ab, ORF3a, ORF7, ORF8, ORF9 and ORF10 play a critical rule in adhesion of virus to host cell and can compromise vaccine efficacy [3]. SARS-CoV-2 shares genetic homology with other coronaviruses found in bats and its closest related human virus, SARS-CoV-1. The spike protein of SARS-CoV-2 has high identity with that of SARS and MERS, which might indicate the similarity of immune evasion mechanism. After publication of the full RNA genetic sequence of COVID-19 from infected patients by Chinese researchers on January 10, 2020 [2], many organizations around the world started to develop vaccines, based on knowledge obtained from SARS and MERS vaccine development, by different means including inactivated whole COVID-19 virus [4–6], live attenuated virus, adenovirus-based recombinant vector RNA and DNA vaccines [Fig. 1]. As of August 24, 2020, WHO documented a total of 160 vaccine candidates against COVID-19, with 26 vaccines currently in clinical evaluation (Table 1) and 137 under pre-clinical evaluation [7]. In order to get herd immunity, an estimated 67% of population needs to be vaccinated to stop the virus spreading [8]. A vaccine targeting the Spike protein receptor-binding domain (S-RBD) of SARS-CoV-2 induces protective immunity [9] in phase II/III human evaluation, after safety and efficacy results in rhesus macaque [10]. Meanwhile, the Ad5 vectored COVID-19 vaccine targeting the spike glycoprotein showed tolerability and immunogenicity at 28 days post-vaccination (NCT04313127) [11]. A few recent studies demonstrated promising results. The Ad5-vectored COVID-19 vaccine at 5 × 10^10^ viral particles was safe, and induced significant immune responses in the majority of recipients after a single immunization (NCT04341389) [12]. Analysis of 2 randomized phase 1 and phase 2 clinical trials of inactivated vaccine showed that patients had a low rate of adverse reactions and demonstrated immunogenicity (ChiCTR2000031809) [13]. Phase 1/2 single-blind, randomised controlled trial with adenovirus vaccine that expresses the spike protein of SARS-CoV-2 in chimpanzee (ChAdOx1 nCoV-19) showed an acceptable safety profile, and homologous boosting increased antibody responses [14]. Meanwhile clinical trial of mRNA-1273 vaccine results showed vaccination of nonhuman primates induced robust SARS-CoV-2 neutralizing activity, rapid protection in the upper and lower airways, and no pathologic changes in the lung [15, 16]. Another mRNA-based vaccine BNT162 was initiated phase I/II trial in China (ChiCTR2000034825).

**Fig. 1:**
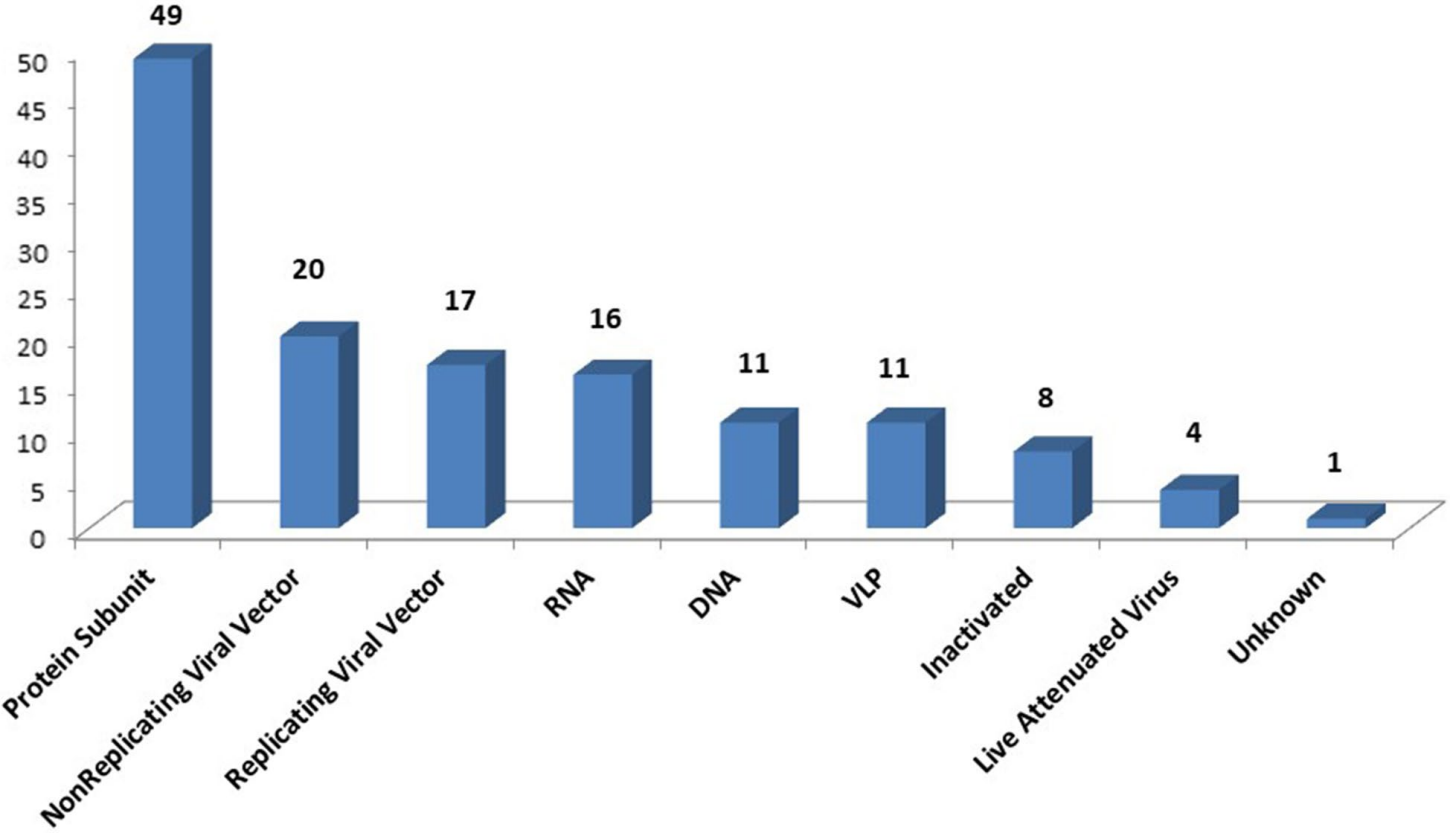
Distribution of COVID-19 vaccine types under development. Data modified from the WHO website: https://www.who.int/blueprint/priority-diseases/key-action/novel-coronavirus-landscape-ncov.pdf

**Table 1:** 26 candidate vaccines in clinical evaluation

Platform	Type of candidate vaccine	Developer	Coronavirus target	Current stage of clinical evaluation/regulatory status coronavirus candidate	Estimated enrollment	Same platform for non-Coronavirus candidates	Trial start date	Estimated completion date
DNA	DNA plasmid vaccine with electroporation	Inovio Pharmaceuticals	INO-4800, Spike glycoprotein of SARS-CoV-2	Phase 1/2 NCT04447781 NCT04336410	160 120	multiple candidates	June 22, 2020 April 3, 2020	February 22, 2022 July 2021
DNA	DNA plasmid vaccine + Adjuvant	Osaka University/ AnGes/ Takara Bio	DNA vaccine (AG0301-COVID19)	Phase 1/2 NCT04463472	30		June 29, 2020	July 31, 2021
DNA	DNA plasmid vaccine	Cadila Healthcare Limited	DNA COVID-19	Phase 1/2 CTRI/2020/07/026352	1048		July 1, 2020	N/A
DNA	DNA Vaccine (GX-19)	Genexine Consortium	DNA COVID-19	Phase 1 NCT04445389	210		June 17, 2020	June 17, 2022
Inactivated	Inactivated + alum	Sinovac	Inactivated COVID-19 virus	Phase 3 NCT04456595 Phase 1/2 NCT04383574 NCT04352608	8870 422 744	SARS	July 2020 May 20, 2020 April 16, 2020	October 2021 July 20, 2020 December 13, 2020
Inactivated	Inactivated	Wuhan institute of Biological Products/Sinopharm	Inactivated COVID-19 virus	Phase 1/2 ChiCTR2000031809	1456		April 11, 2020	November 10, 2021
Inactivated	Inactivated	Beijing Institute of Biological Products/Sinopharm	Inactivated COVID-19 virus	Phase 1/2 ChiCTR2000032459	1456		April 28, 2020	November 28, 2021
Inactivated	Whole-Virion Inactivated	Bharat Biotech	Inactivated COVID-19 virus	Phase 1/2 CTRI/2020/07/026300	1125			
Inactivated	Inactivated	Institute of Medical Biology, Chinese Academy of Medical Sciences	Inactivated COVID-19	Phase 1 NCT04412538	942		May 15, 2020	September, 2021
NonReplicating Viral Vector	ChAdOx1	University of Oxford/AstraZeneca/Serum Institute of India	Recombinant COVID-19 (chimpanzee adenovirus vector ChAdOx1)	Phase 3 ISRCTN89951424 Phase2b/3 EUCTR2020-001228–32-GB Phase 1/2 PACTR2020069221	2000 12,330 2000	MERS, influenza, TB, Chikungunya, Zika, MenB, plague	May 01, 2020 June 24, 2020	July 31, 2021 December 30, 2021
NonReplicating Viral Vector	Adenovirus Type 5 Vector	CanSino Biological Inc./Beijing Institute of Biotechnology	Recombinant COVID-19 (Adenovirus Vector)	Phase 2 ChiCTR2000031781 Phase 1 ChiCTR2000030906	500 108	Ebola	April 12, 2020 March 16, 2020	January 31, 2021 December 31, 2020
NonReplicating Viral Vector	Adeno-based	Gamaleya Research Institute	Recombinant COVID-19 adenovirus vector	Phase 1 NCT04436471 NCT04437875	38 38		June 17, 2020	August 15, 2020
Protein	Recombinant Novel Coronavirus Vaccine (Adenovirus Vector)	Hubei Provincial CDC	Recombinant COVID-19 (Adenovirus Vector)	Phase II NCT04341389	508		April 12, 2020	January 31, 2021
Protein	Adenovirus Type 5 Vector	Hubei Provincial CDC	Recombinant COVID-19 (Adenovirus Type 5 Vector)	Phase I NCT04313127	108		March 15, 2020	December 30, 2020
Protein Subunit	Full length recombinant SARs CoV-2 glycoprotein nanoparticle vaccine adjuvanted with Matrix M	Novavax	SARS-CoV-2 rS (COVID-19) nanoparticle	Phase 1/2 NCT04368988	131	RSV; CCHF, HPV, VZV, EBOV	May 25, 2020	July 31, 2021
Protein Subunit	Native like Trimeric subunit Spike Protein vaccine	Clover Biopharmaceuticals Inc./GSK/Dynavax	Recombinant SARS-CoV-2 trimeric s protein subunit vaccine for COVID-19	Phase 1 NCT04405908	150	HIV, REV Influenza	June 19, 2020	March 30, 2021
Protein Subunit	Adjuvanted recombinant protein (RBDDimer)	Anhui Zhifei Longcom Biopharmaceutical/ Institute of Microbiology, Chinese Academy of Sciences	Adjuvanted recombinant protein (RBDDimer) (CHO Cells)	Phase 1 NCT04445194	50	MERS	June 22, 2020	September 20, 2021
Protein Subunit	Recombinant spike protein with Advax™ adjuvant	Vaxine Pty Ltd/Medytox	Recombinant spike protein	Phase 1 NCT04453852	40		June 30, 2020	July 1, 2021
Protein Subunit	Molecular clamp stabilized Spike protein	University of Queensland/GSK/Dynavax	Molecular clamp stabilized Spike protein	Phase 1 ACTRN12620000674932p	120	Nipah, influenza, Ebola, Lassa		
RNA	LNP-encapsulated mRNA	Moderna NIAID	mRNA-1273 COVID-19	Phase 2 NCT04405076 Phase 1 NCT04283461	600 120	multiple candidates	May 29, 2020 March 16, 2020	August, 2021 November 22, 2021
RNA	3 LNP-mRNAs	biotech/Fosum Pharma/Pfizer	RNA COVID-19	Phase ½ EUCTR2020-001038–36-DE NCT04368728	444 32,000		April 20, 2020 April 29, 2020	January 23, 2023
RNA	LNP-nCoVsaRNA	Imperial College London	LNP-nCoVsaRNA	Phase 1 ISRCTN17072692	320	EBOV; LASV, MARV, Inf (H7N9), RABV	April, 2020	July, 2021
RNA	mRNA	Curevac	mRNA Vaccine CVnCoV	Phase 1 NCT04449276	168	RABV, LASV, YFV; MERS, InfA, ZIKV, DENV, NIPV	June 18, 2020	August, 2021
RNA	mRNA	People's Liberation Army (PLA) Academy of Military Sciences/Walvax Biotech	mRNA COVID-19	Phase 1 ChiCTR2000034112	168		June 25, 2020	December 31, 2021
RNA	mRNA	Jiangsu Provincial CDC	SARS-CoV-2 mRNA vaccine (BNT162b1)	Phase I ChiCTR2000034825	144		July 20, 2020	December 31, 2020
VLP	Plant-derived VLP adjuvanted with GSK or Dynavax adjs	Medicago Inc	Coronavirus-like particle COVID-19	Phase 1 NCT04450004	180	Flu, Rotavirus, Norovirus, West Nile virus, Cancer	July 10, 2020	April 30, 2021

Data modified from the WHO website: https://www.who.int/blueprint/priority-diseases/key-action/novel-coronavirus-landscape-ncov.pdf

Both live-attenuated vaccines and inactivated vaccines are highly established in product development and manufacturing process but require handling live virus. Meanwhile recombinant protein-based and vector-based vaccines are safe but require epitope selection, antigen design, and vehicle development. Some new-generation vaccine types were not produced on large scale before. RNA and DNA vaccines are two new vaccine technologies currently in focus for COVID-19 vaccine development.

Vaccine development for COVID-19 is a highly complex process involving viral genomic studies, identification of target for vaccine, vaccine design, manufacturing, storage and distribution, preclinical and clinical safety and efficacy studies. The high levels of efforts and global collaboration at this scale is unprecedented. Due to the special nature of this novel virus, vaccine development for COVID-19 seems to be very challenging. However, with the accumulation of more knowledge about the virus and the efforts of global scientific cooperation, the covid-19 vaccine will be successfully developed, and the COVID-19 pandemic will eventually be controlled.

## Data Availability

Not Applicable.

## Abbreviations

COVID-19: Corona virus 2019
WHO: World health organization
SARS: Severe acute respiratory syndrome
MERS: Middle East Respiratory Syndrome
